# Supplementary material for: Promoter-proximal pausing mediated by the exon junction complex regulates splicing
Source: Nat Commun. 2019 Jan 31;10:521. doi: 10.1038/s41467-019-08381-0 (PMC6355915; doi:10.1038/s41467-019-08381-0)
Supplement: Supplementary file 1 — Supplementary Information [file 41467_2019_8381_MOESM1_ESM.pdf]

# Supplementary Information Files

## Supplementary Figure 1

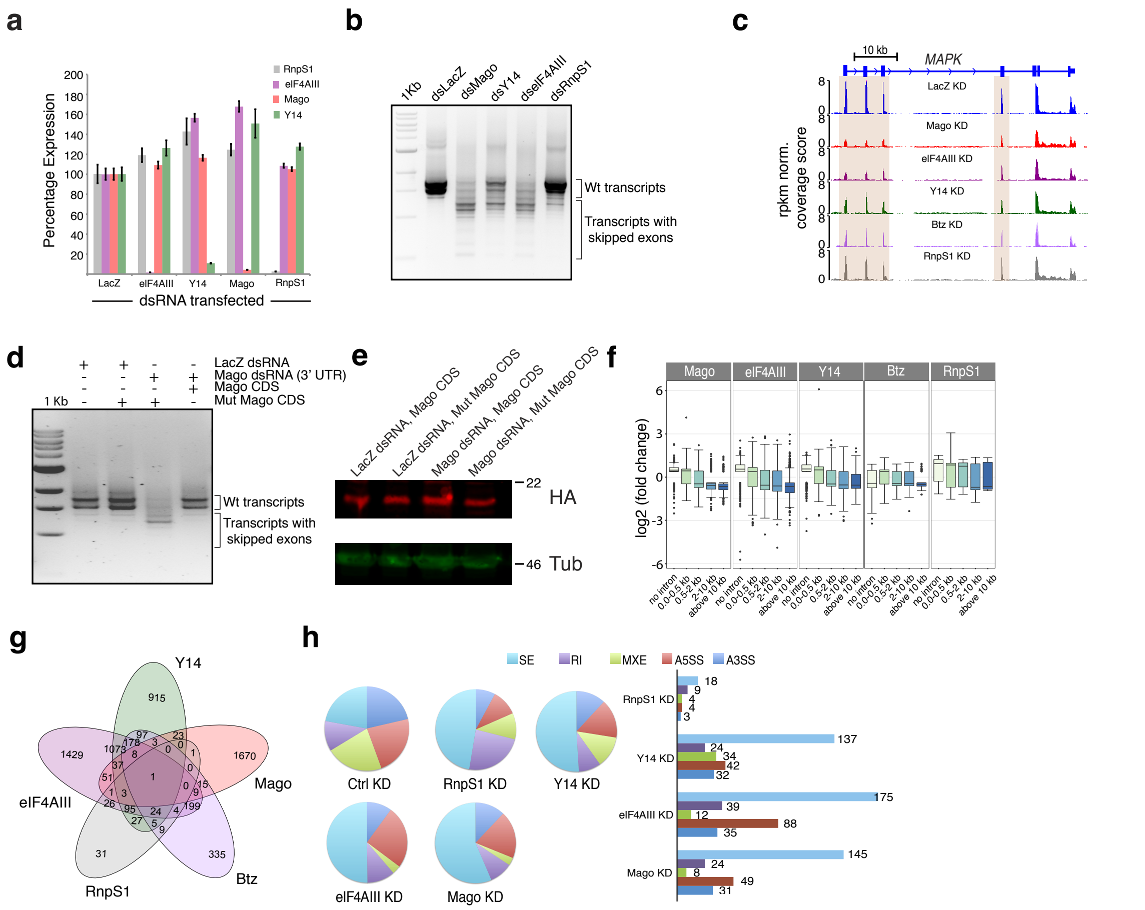


**The pre-EJC affects expression and splicing of large intron-containing transcripts**
**(a)** RT-qPCR analysis showing the efficiency of knockdown for the indicated conditions. **(b)** Agarose gel of semi-quantitative RT-PCR for *MAPK* transcripts using RNA from S2R+ cells in the indicated knockdowns. Note that the depletion of pre-EJC core components (Mago, Y14, and elF4AIII) has greater effect on exon definition than RnpS1. **(c)** Genome browser view of averaged steady state RNA-Seq data for *MAPK* transcript from S2R+ cells in the indicated knockdowns. Reads per million are shown on Y-axis. The skipped exons are highlighted with colored rectangle. **(d)** Agarose gel of semi-quantitative RT-PCR for *MAPK* transcripts using RNA from S2R+ cells with dsRNA targeting the 3’ UTR of Mago. The knockdown was performed in S2R+ cells either transfected with the wild type Mago CDS or mutant Mago (EJC interaction mutant) CDS without 3’ UTR. Note that knockdown in cells transfected with wild type Mago CDS does not lead to splicing defects in *MAPK,* however the mutant Mago fails to rescue the splicing defect. The primers used for the PCR anneals in the 5’ and 3’ UTR of *MAPK*, as described before. **(e)** Western blot using antibody directed against HA, showing expression of wild type and mutant Mago in S2R+ cells. Shown below is the western blot against Tubulin, used as loading control. **(f)** Log2 fold changes in steady state RNA levels in indicated knockdowns compared to control knockdown, when genes are separated according to the size of their largest introns. The Y-axis shows the fold changes in expression while the X-axis depicts different classes. **(g)** Venn diagram showing the overlap between differentially expressed genes in indicated knock down conditions. **(h)** rMATS analysis of steady state RNA-Seq from S2R+ cells in the indicated knockdowns. (Left) The pie-charts depicts the percentage of significant events with skipped exons (SE), retained introns (RI), mutually exclusive exons (MXE),

alternative 5’ splice site (A5SS) and alternative 3’ splice site (A3SS) in each condition, along the control with proportion of all events. (Right) Histograms showing the number of these events in indicated knockdowns when compared to control.

## Supplementary Figure 2

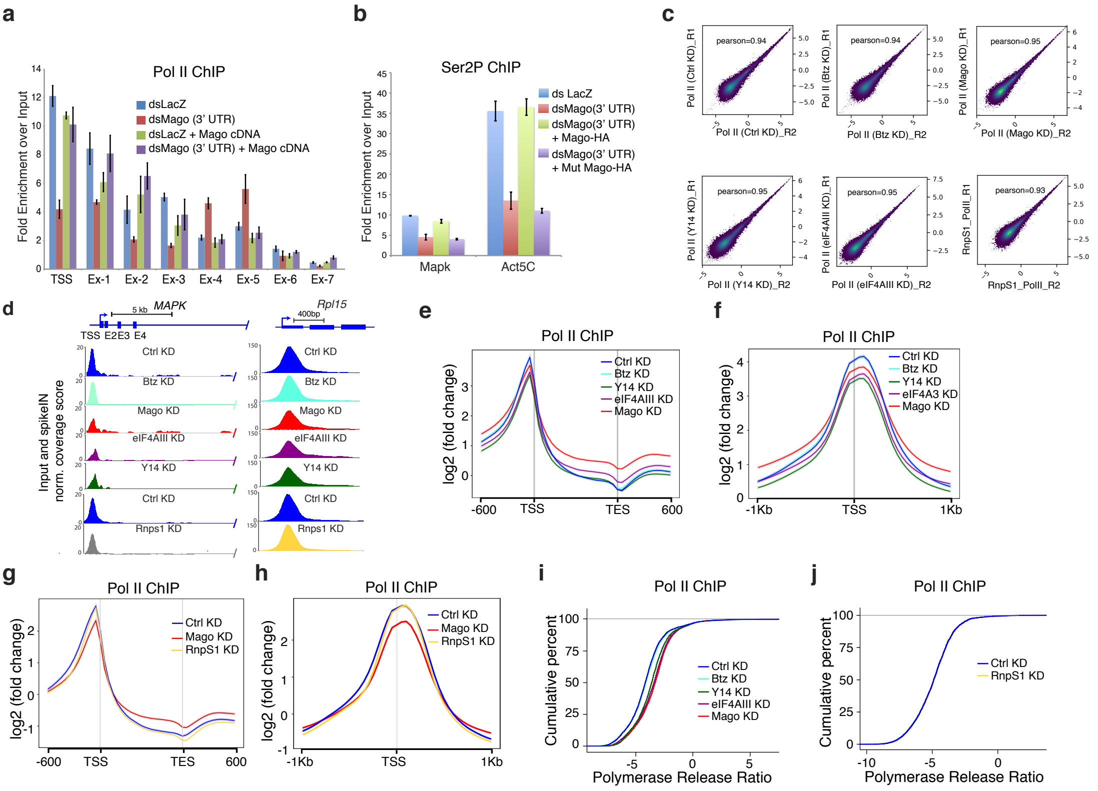


**The EJC modulates promoter proximal pausing independently of Btz and of its splicing subunit RnpS1**

**(a)** ChIP-qPCR analysis of Pol II occupancies at *MAPK* locus. The tested regions for enrichment are same as shown in figure 1A. The knockdown was performed in S2R+ cells with dsRNA targeting the 3' UTR of Mago, either transfected with a control CDS or Mago CDS without 3' UTR. Note that knockdown in the cells transfected with Mago CDS does not lead to change in Pol II occupancies. Error bars indicate the standard deviation from the mean in three biological replicates. **(b)** ChIP-qPCR analysis of Ser2P rescue at the indicated loci with either wild type Mago or EJC interaction mutant Mago. The knockdown was performed in S2R+ cells with dsRNA targeting the 3' UTR of Mago, either transfected with a control CDS or mutant Mago CDS without 3' UTR. Note that cells transfected with wild type Mago CDS rescues Ser2P level to the control condition, while mutant Mago CDS does not rescue the Ser2P level. Error bars indicate the standard deviation from the mean in three biological replicates. **(c)** Pearson correlation between the replicates of Pol II ChIP-Seq for indicated knockdowns**.** Each dot represent individual bin in the genome at which the correlation in signal intensity between each replicates was calculated (indicated on X and Y axis) **(d)** Input and "spike-in" normalized and replicate averaged track examples of total Pol II enrichment of ChIP-Seq from S2R+ cells extracts, after either control or indicated knockdowns. Shown here are *MAPK* and Rpl15 loci. **(e)** Metagene profiles based on averaged total Pol II occupancy of two independent biological replicates with "spike-in" normalization in control and indicated knockdown conditions with standard error of mean for all the expressed genes. Log2 fold changes against input control are shown on Y-axis, while X-axis depicts scaled genomic coordinates. **(f)** Replicate averaged metagene profiles of total Pol II occupancies in control and in indicated knockdown conditions with standard error of mean for all the expressed genes, after ""spike-in"" normalization, centered at the TSS in a ± 1 Kb window. Log2 fold changes against input control are shown on Y-axis, while X-axis shows genomic coordinates. **(g)** Metagene profiles based on the average total Pol II occupancy of two independent biological replicates with "spike-in" normalization in control, Mago and RnpS1 knockdown conditions with standard error of mean for all the

expressed genes. Log2 fold changes against input control are shown on Y-axis, while X-axis depicts scaled genomic coordinates. **(h)** Metagene profiles based on the average of two independent biological replicates of total Pol II occupancies in control, Mago and RnpS1 knockdowns with standard error of mean for all the expressed genes, after “spike-in” normalization, centered at the TSS in a ± 1 Kb window. Log2 fold changes against input control are shown on Y-axis, while X-axis shows genomic coordinates. **(i)** ECDF plots of PRR in cells with control and indicated knockdowns. KD of pre-EJC components has substantial effect on PRR while KD of the cytoplasmic component Btz has no effect. **(j)** ECDF plots of PRR in cells with control and RnpS1 knockdowns. The RnpS1 knockdown has no effect on the PRR.

**Supplementary Figure 3**

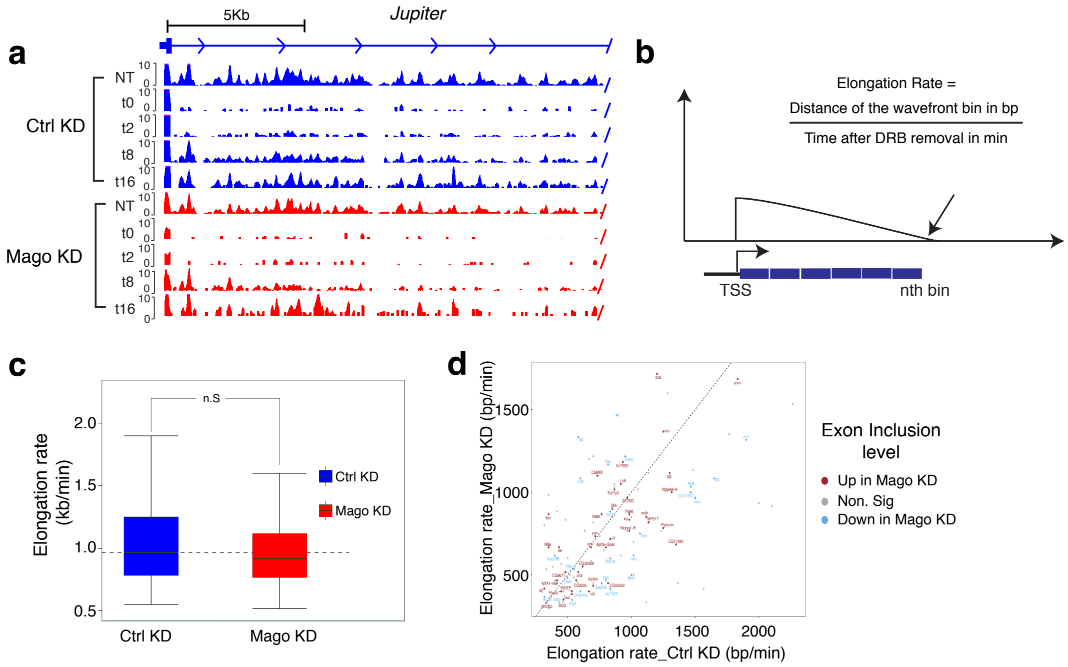


**Mago does not influence the elongation rate**

**(a)** Genome browser view of DRB-4sU-Seq in control or Mago-depleted cells, shown here is *jupiter*. Time points indicate the time (in minutes) for which transcription was allowed to continue after removal of DRB. **(b)** Schematic representation of the calculation of the elongation rate. Genes longer than 10 kb were divided into 100 bp bins and the transcriptional wave front was identified in the bin with lowest local minima signal. The distance covered by the wavefront between 2 min after DRB removal and 8 min is then divided by the corresponding time interval (8 – 2 min) to calculate elongation rates. **(c)** Box plots showing the distribution of elongation rate in control and Mago-depleted S2R+ cells. **(d)** Scatterplot showing the relationship between elongation rate and exon inclusion level. The exon of the genes with the highest difference in inclusion level between control and Mago KD was considered for analysis (DEXseq was used for inclusion level estimates). The red and blue dots depict the genes with inclusion level in Mago knockdown higher and lower respectively, when compared to control knockdown. The grey dot depicts all those genes where the difference in inclusion level between the two conditions was not significant.

**Supplementary Figure 4**

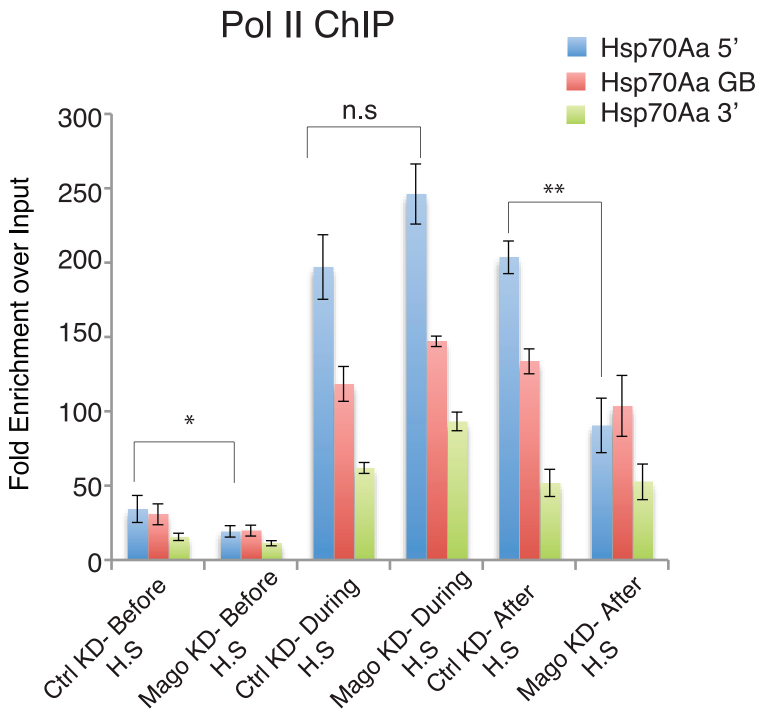


**Mago maintains Pol II pausing at *heat shock protein* gene**

ChIP-qPCR analysis of Pol II occupancies at *Hsp70Aa gene* before, during and after heat shock treatment in either control or mago depleted S2R+ cells. The regions tested for enrichment are promoter, gene body, and 3' end of the *Hsp70Aa* gene.

**Supplementary Figure 5**

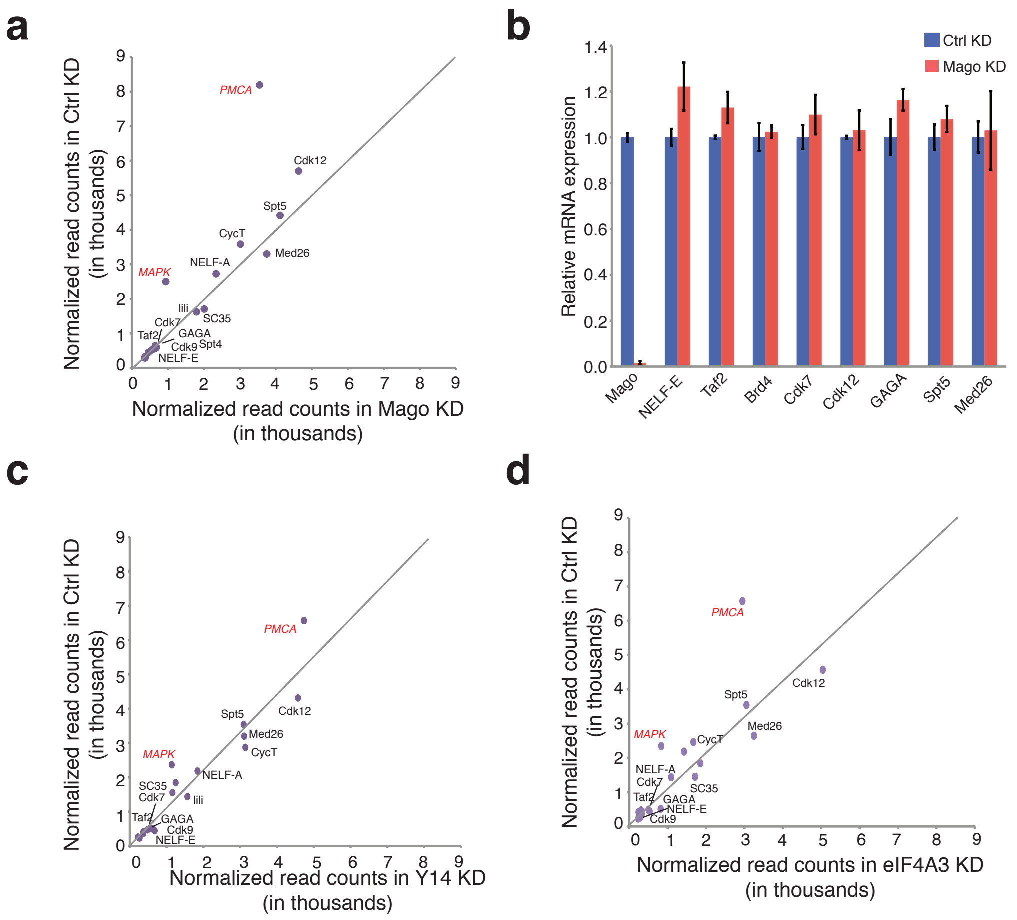


**Pre-EJC components do not control the expression of paused and elongation factors, but bind nascent RNA**

**(a)** Scatterplot showing the normalized read counts from DESeq2 of well-characterized elongation and paused factors in control and Mago-depleted S2R+ cells. Two Mago targets (*MAPK* and *PMCA*) are shown in red as controls. **(b)** Quantitative RT-PCR showing the transcript levels of genes involved in elongation and pause release control, using RNA extracts derived from S2R+ cells upon control and Mago knockdowns. Error bars indicate the standard deviation from the mean of two biological replicates. **(c, d)** Scatterplot showing the normalized read counts from DESeq2 of well-characterized elongation and paused factors in control, Y14 **(c)** and eIF4AIII **(d)** depleted S2R+ cells. Two of the pre-EJC targets (*MAPK* and *PMCA*) are shown in red as controls.

## Supplementary Figure 6

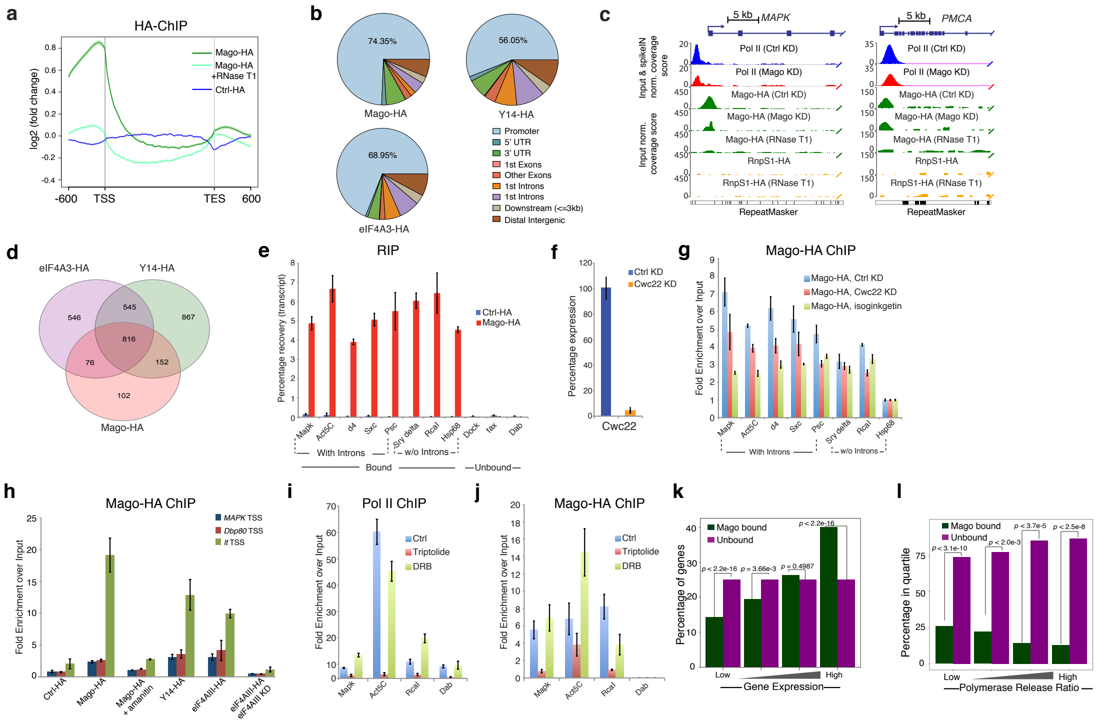


**Pre-EJC gets preferentially recruited to highly expressed and low PRR genes via binding to nascent RNA**

**(a)** Metagene profile of ChIP-Seq performed with HA-tagged Mago and Ctrl HA-tag in the presence or absence of RNase T1, with standard error of mean for all the expressed genes (based on the average enrichment of two replicates). Log2 fold changes against input control are shown on Y-axis while X-axis depicts scaled genomic coordinates. **(b)** Pie charts showing the distribution of pre-EJC component binding. **(c)** Input normalized and replicate averaged track examples of ChIP-Seq experiments from S2R+ cell extracts transfected with HA-tagged Mago or HA-tagged RnpS1. The cells were either subjected to control or Mago knockdown and chromatins were either untreated or treated with RNase T1, as indicated. Shown here are two pre-EJC target genes *MAPK* and *PMCA*. **(d)** Venn diagram showing the overlap between genes bound by pre-EJC components. **(e)** RT–qPCR quantification depicting percentage recovery of Mago-bound transcripts in RNA immunoprecipitation with HA tagged Mago, compared to the control-HA tag. Error bars represent the standard deviation from three independent biological replicates. **(f)** RT-qPCR analysis showing the efficiency of Cwc22 knockdown. **(g)** ChIP-qPCR experiments showing recruitment of Mago at the TSS of indicated genes, in control and Cwc22 knockdown conditions, as well after treating the cells with a splicing inhibitor (isoginkgetin) for six hours. Note that Mago recruitment was resistant to Cwc22 KD and only mildly affected with the drug treatment. Error bars indicate the standard deviation from the mean of two biological replicates. **(h)** ChIP-qPCR experiments showing recruitment of pre-EJC components (Mago, Y14, and elF4AIII) at the TSS of indicated genes. Note that Mago recruitment was impaired when transcription was blocked using α-amanitin. Error bars indicate the standard deviation from the mean of two biological replicates. **(i)** ChIP-qPCR experiments showing recruitment of Mago at the TSS of indicated genes, in control and as well after treating the cells with a transcription initiation inhibitor (Triptolide) and with elongation inhibitor (DRB) for six hours. Note that Mago recruitment was resistant to DRB treatment, however treatment with triptolide results in loss of Mago binding. Error bars indicate the standard deviation from the mean of two biological replicates. **(j)** ChIP-qPCR experiments showing Pol II occupancies at the TSS of indicated genes after the indicated drug treatment. Note that as expected triptolide leads to loss of Pol II

occupancies while DRB had no significant effect as described earlier, showing the efficacy of the drug treatment. Error bars indicate the standard deviation from the mean of two biological replicates. **(k)** Histogram showing percentage of Mago-bound genes amongst different quartiles of genes expression, for genes expressed in control condition. For quartile classification, all of the expressed genes in S2R+ cells were divided into four equal sized quartiles according to the level of their expression, from low to high level. *P*-values for significance of the enrichment for Mago binding amongst different quartiles, derived from Fisher's exact test, are shown on top of the histogram. **(l)** Histogram showing percentage of genes bound by Mago in different quartiles of PRRs. For quartile classification, all of the expressed genes in S2R+ cells were divided into four equal sized quartiles according to the calculated PRRs, from low to high level. *P*-values for significance of the associations, derived from Fisher's exact test, are shown on top of the histogram.

**Supplementary Figure 7**

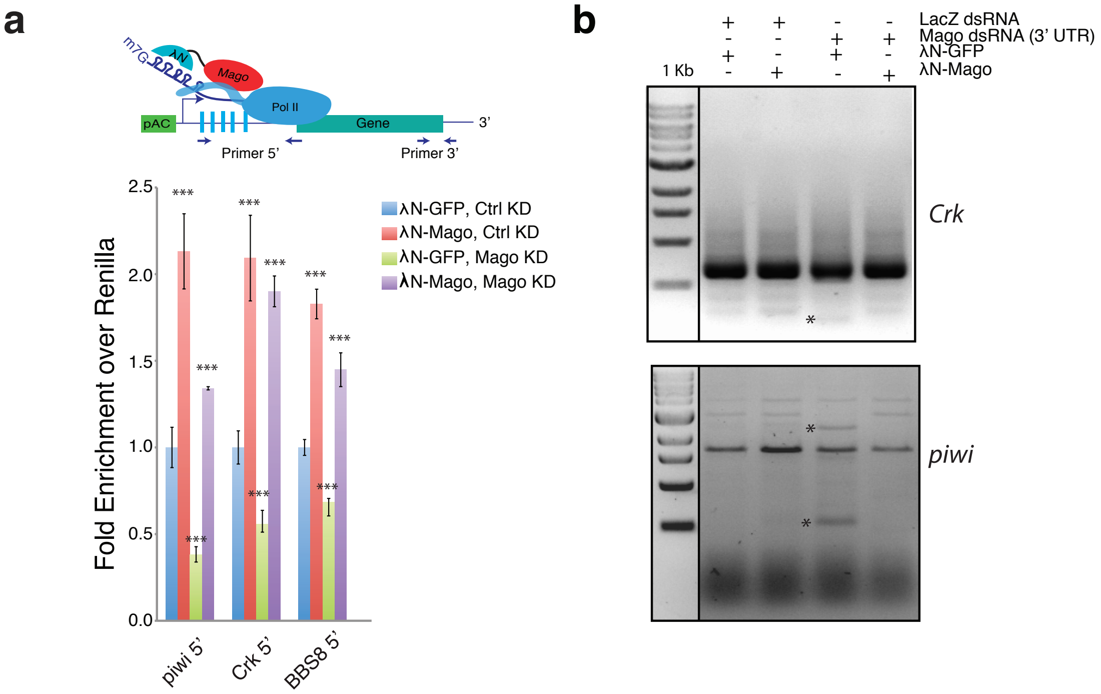


**Tethering Mago to 5'UTRs increases Pol II pausing and rescues splicing defects.**
**(a)** Recruitment of Mago at the 5' end of RNA is sufficient to induce pausing. (Top) Schematic of the BoxB-λN tethering assay. BoxB sequences (blue rectangles) were inserted upstream of the CDS of indicated genes (green rectangle). The λN peptide (blue) was fused to Mago (shown in red) or GFP, and transfected into S2R+ cells along with a Renilla luciferase construct. (Bottom) Quantification of the ChIP experiment. Chromatin was prepared for the different conditions and followed by immunoprecipitation using antibody directed against total Pol II. The enrichment of Pol II at the promoter and at the 3' end of indicated genes was calculated after normalizing against a negative loci and Renilla. The enrichment for three independent biological replicates is shown along with *P*-values, for tested conditions. **(b)** Agarose gel of semi-quantitative RT-PCR for *piwi* and *Crk* transcripts using RNA from S2R+ cells in the indicated conditions. Note that tethering Mago to 5' end of the gene rescues the observed splicing defects, while tethering GFP has no effect.

## Supplementary Figure 8

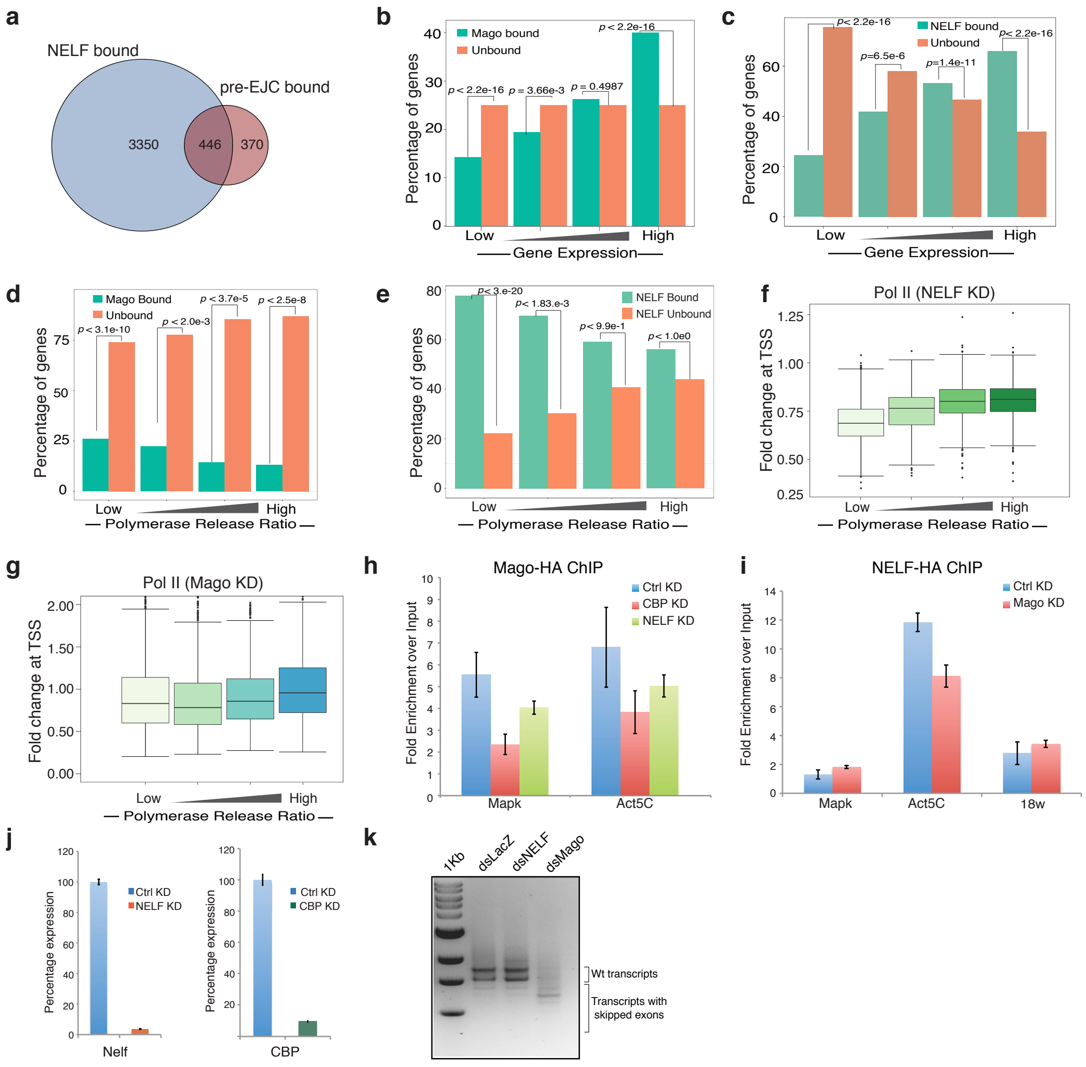


**Comparison of Mago- and NELF-mediated Pol II pausing**

**(a)** Venn diagram showing the overlap between genes bound by pre-EJC components and NELF[1]. **(b, c)** Histogram showing percentage of pre-EJC bound genes (b) and NELF (c) amongst different quartiles of genes expressed in control condition. For quartile classification, all of the expressed genes in S2R+ cells were divided into four equal sized quartiles according to the level of expression, from low to high level. *P*-values for significance of the associations, derived from Fisher's exact test, are shown on top of the histogram. **(d, e)** Histogram showing percentage of genes bound by Mago (d) and NELF (e) in different quartiles of PRRs. For quartile classification, all of the expressed genes in S2R+ cells were divided into four equal sized quartiles according to the calculated PRRs, from low to high level. **(f, g)** Fold changes in Pol II occupancies in Mago (f) and NELF (g) knock down conditions at the promoter (TSS ± 250bp). For quartile classification, all of the expressed genes in S2R+ cells were divided into four equal sized quartiles according to the calculated PRRs, from low to high level. **(h)** ChIP-qPCR experiments showing Mago-HA occupancies at the TSS of *MAPK* and *Act5C* after the indicated knockdowns. **(i)** ChIP-qPCR experiments showing NELF-HA occupancies at the TSS of *MAPK* and *Act5C* after Mago knockdown. **(j)** RT-qPCR analysis showing the efficiency of knockdown for the indicated conditions. **(k)** Agarose gel of semi-quantitative RT-PCR for *MAPK* transcripts using RNA from S2R+ cells in the indicated knockdowns. Note that the depletion of Mago leads to exon skipping at MAPK locus while NELF KD has no effect.

**Supplementary Figure 9**

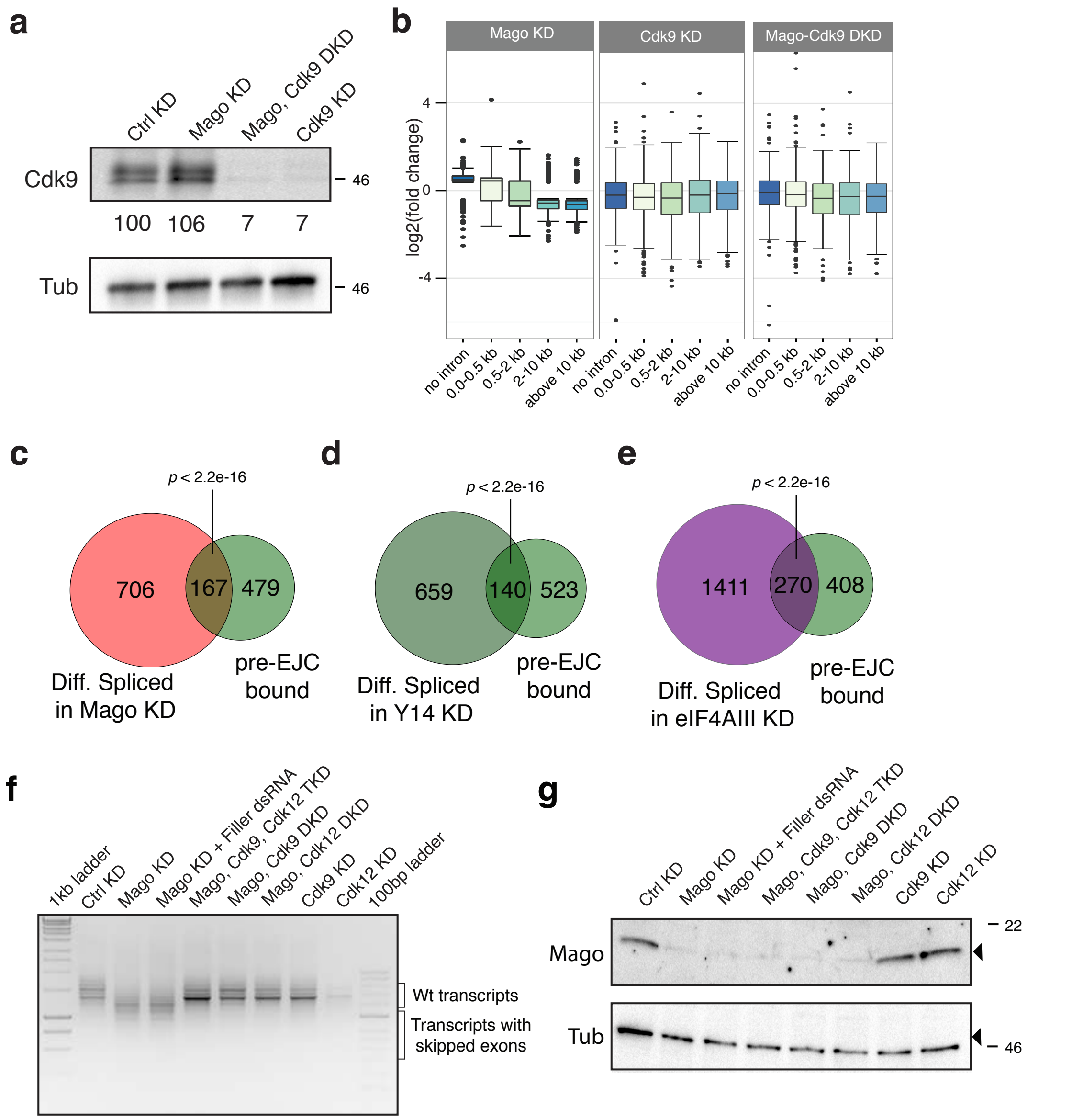


**Intron length-dependent changes upon Mago knockdown can by rescued by simultaneous co-depletion of Cdk9 or Cdk12**

**(a)** Western blot using antibody directed against Cdk9, showing expression and knockdown efficiency of Cdk9 in S2R+ cells in indicated conditions. Signal in the knockdown conditions was normalized to the control condition using Tubulin as loading control and quantification of the intensity was performed with ImageJ. **(b)** Depletion of Mago in S2R+ cells results in differential gene expression in an intron size dependent manner, when compared to the control condition. Double knockdown of Mago and Cdk9 results in loss of the size dependency effect on gene expression. Shown also is Cdk9 knockdown which affects gene expression independently of intron size, when compared to the control condition. **(c-e)** Venn diagrams showing the overlap between pre-EJC bound genes and genes with differential splicing upon pre-EJC components KD. The overlaps between genes that are differentially spliced upon pre-EJC components KD, and are simultaneously bound by pre-EJC are significant. *P*-values are derived from Fisher's exact test. **(f)** Agarose gels showing RT-PCR products for *MAPK* using RNA extracted from S2R+ cells with indicated knockdowns as template for cDNA synthesis. The primers used span the 5' and 3' UTR of *MAPK*, as described before. Note that similar to Mago and Cdk9 double

knockdown, co-depletion of Cdk12 and Mago also rescues *MAPK* splicing defects. The triple knockdown of Mago, Cdk9, and Cdk12 has a slightly better rescue of *MAPK* splicing defects than either of the double knockdowns. **(g)** Western blot using antibody directed against Mago, showing expression and knockdown efficiency of Mago in S2R+ cells in indicated conditions. Shown below is the western blot against Tubulin, used as loading control.

**Supplementary Figure 10**

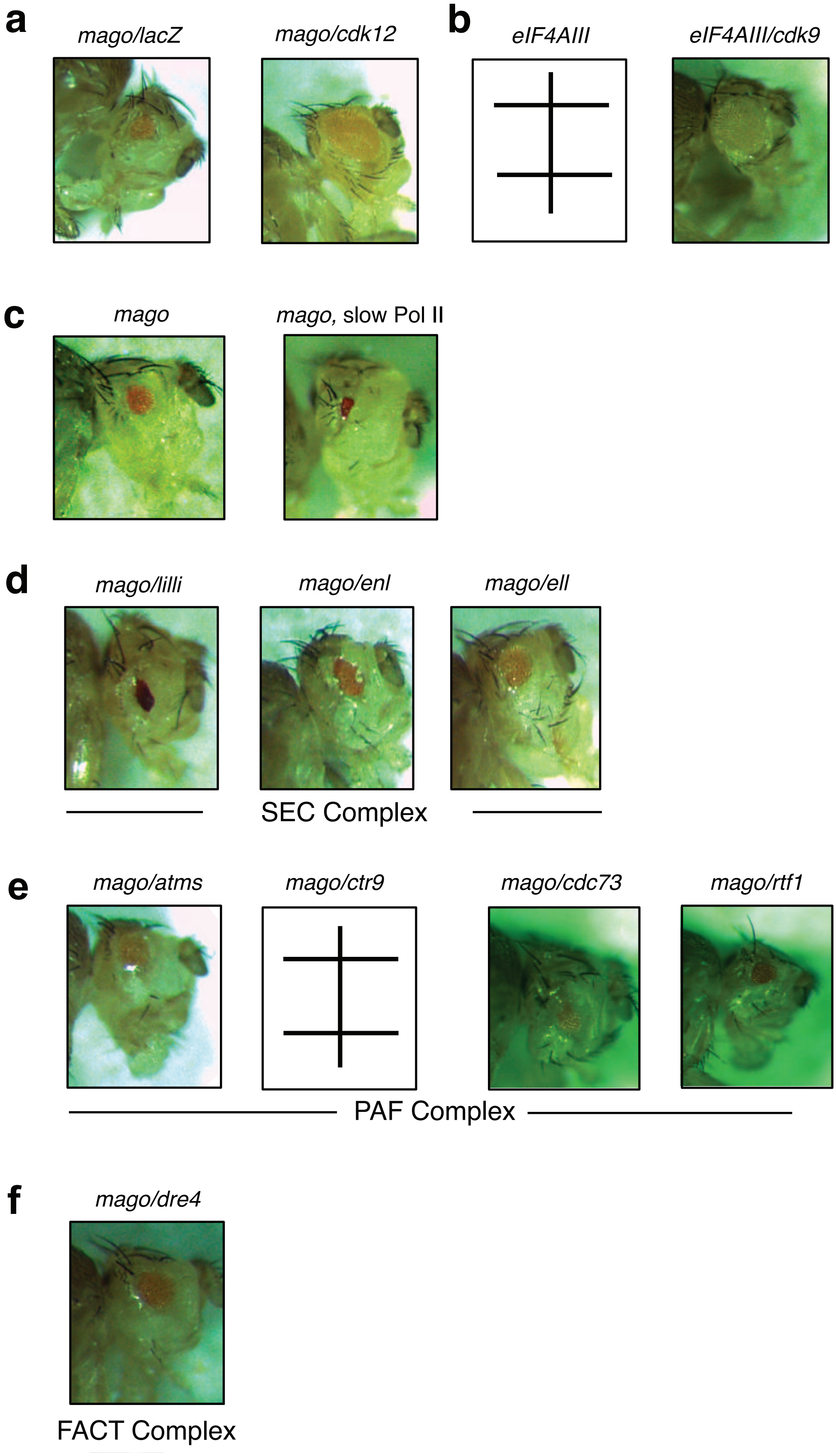

**Depletion of elongation factors or slowing down RNA Pol II does not rescue Mago photoreceptor differentiation defects (a-f)**

*Drosophila* adult eyes in different conditions. **(a)** Loss of Mago in the eye results in impairment of eye development due to lack of photoreceptor differentiation. Simultaneous depletion of Cdk12 restores photoreceptor differentiation. **(b)** Loss of eIF4AIII in the eye results in lethality. Simultaneous depletion of Cdk9 restores viability and photoreceptor differentiation. **(c)** Slowing down the kinetics of Pol II using a slow *Pol II* mutant fails to rescue Mago's effect on photoreceptor differentiation. **(d-f)** Knockdowns of SEC complex components **(d)**, PAF complex components **(e)** or the Dre4 FACT complex subunit **(f)**, do not substantially rescue the eye phenotype resulting from Mago depletion.

# Supplementary Figure 11: Uncropped blot images with molecular weight

Fig 2d

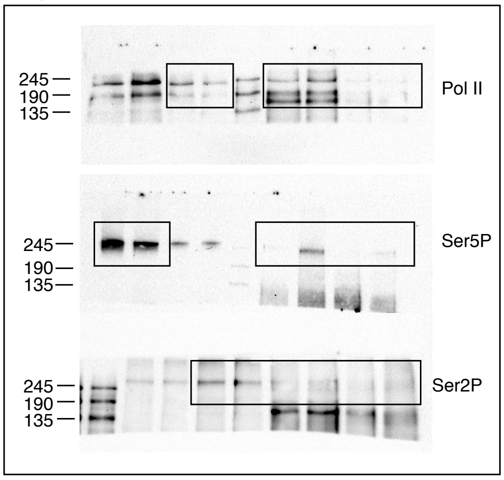


Fig 5f

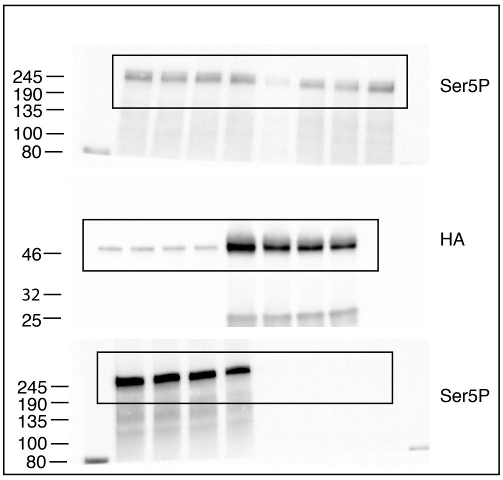


Fig 6a

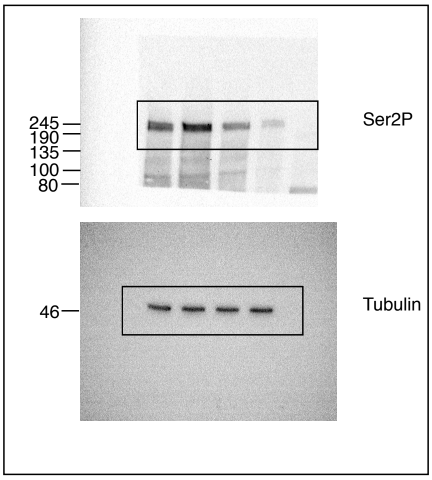


Fig 7e

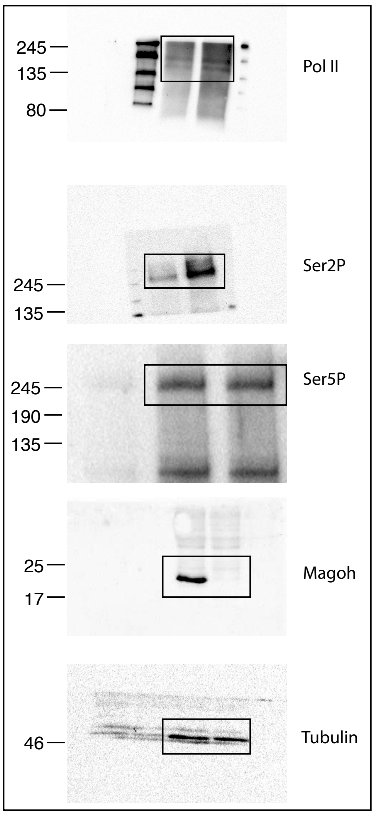

Fig 7f

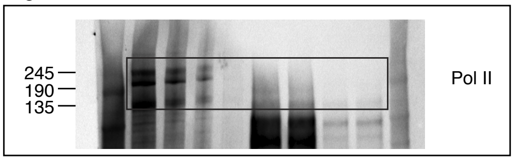


Fig 7g

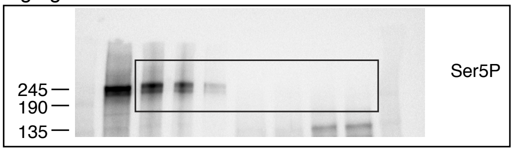


Fig 7h

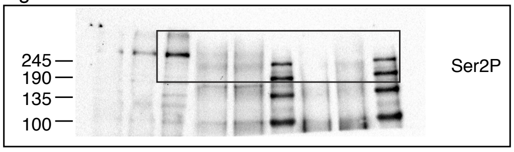


Fig 7i

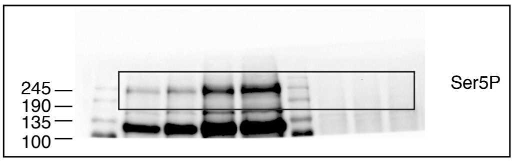


Fig S9a

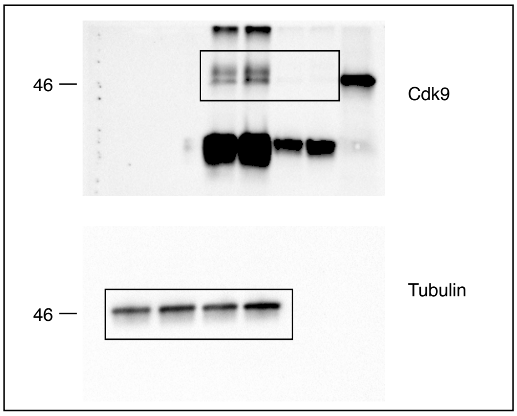


Fig S9G

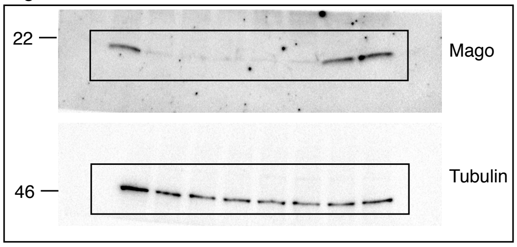

Supplementary_Table1: Primers used in this study

Primers used for the Knockdown experiments

| | |
|---|---|
| Mago T7-Fwd | TAATACGACTCACTATAGGGATTCCGTGTGTTTTCCCATCTCTAAC |
| Mago T7-Rev | TAATACGACTCACTATAGGGGACATATACGAATTGTACAAATGTAAAGGAC |
| RnPS1-T7 Fwd | TAATACGACTCACTATAGGGCACATCGGACAGCAGCTCTTC |
| RnPS1-T7 Rev | TAATACGACTCACTATAGGGGTGTGGTATTTGCTCACCTTTATTA |
| MagohB RNAi | GATATGCCAACAACAGCAA |
| Magoh SiRNA-1 | CGGGAAGTTAAGATATGCCAA |
| Magoh SiRNA-2 | CAGGCTGTTTGTATATTTAAT |
| LacZ-T7 Fwd | TAATACGACTCACTATAGGGCAGGCTTTCTTTCACAGATG |
| LacZ-T7 Rev | TAATACGACTCACTATAGGGCTGATGTTGAACTGGAAGTC |
| Y14-T7 Fwd | TAATACGACTCACTATAGGGCTGGATACTGTTTGTCACCTCTATCC |
| Y14-T7 Rev | TAATACGACTCACTATAGGGGACATAGAGAGCGAAGACGATACG |
| elF4AIII-T7 Fwd | TAATACGACTCACTATAGGGGGACCTCTCCAACGTGGAGTTCG |
| elF4AIII-T7 Rev | TAATACGACTCACTATAGGGATATCGCCGTGCATCGAGCTGAC |
| Cdk9-T7 Fwd | TAATACGACTCACTATAGGGCTACCAAACCCGGTCAATCATAC |
| Cdk9-T7 Rev | TAATACGACTCACTATAGGGATCCTGCACCGAGACATGAAAG |
| NELF T7 Fwd | TAATACGACTCACTATAGGGCGTCAAGCATTTTACGCTGA |
| NELF T7 Rev | TAATACGACTCACTATAGGGGCTTGATGTCCAGCTTCTCC |
| NELF T7 Fwd-2 | TAATACGACTCACTATAGGGGCCATAAAGACGATGCCACT |
| NELF T7 Rev-2 | TAATACGACTCACTATAGGGGCAGGATCTGCTTTCCAGAC |
| Mago 3' UTR T7 Fwd | TAATACGACTCACTATAGGGGCCGTAGCCACCACCTCAAG |
| Mago 3' UTR T7 Rev | TAATACGACTCACTATAGGGGCAAGTCTCAAAACCGTATGTGAG |

Primers used for the qPCR experiments

| | |
|---|---|
| Mapk Mago ChIP F | GGCTGCCAAAAGACTGATGT |
| Mapk Mago ChIP R | ATCAAAATCGACGGCGTAAA |
| Lt Mago ChIP F | GCAGTTTTGGGAGGTTTGTT |
| Lt Mago ChIP R | CCATTTAAAGCAAACGGAAA |
| Dbp80 Mago ChIP F | CCTCTCAAGCTTTTCCAACC |
| Dbp80 Mago ChIP R | TTCCACATAGCTTCATCCAGAA |
| Y14 qPCR-F | GCGGAACGAAAACGAAATAA |
| Y14 qPCR-R | GAACTCCTCCGCATTGTCA |
| elF4AIII qPCR-F | CTACGATTTGCCCAACAACC |
| elF4AIII qPCR-R | CATCTCGTCGATTTGTGTGG |
| Mago qPCR-F | ACTTATCGATACGCGGTGAT |
| Mago qPCR-R | TGGTTTCCCACTCTTTTTCC |
| Mago human qPCR-F | CCATGTCCACACCAATATTCA |
| Mago human qPCR-R | TCCAGAAGGCTTACGAGTATTTT |
| MagoB human qPCR-F | CTGTCGGCCAACCCTATCAG |
| MagoB human qPCR-R | TGTGCACAAGAGTGTAATGGAA |
| Mago_qPCR Fwd | TCACAAACTGGCAAATTGTCTT |
| Mago_qPCR Rev | TCTCAAAACCGTATGTGAGTGC |
| Cdk12-F | TTCGGGAACTGGTACCTCTG |
| Cdk12-R | CGGCGATGATGATAGTGATG |
| Taf2-F | ATAGCAACGGAGACCACAGG |
| Taf2-R | CGGAAGTGATGCGTTTTCTT |
| Spt-5 F | GTAATCTTCGGATGGGCAAA |
| Spt-5R | TGCGATGTCGTCCTTGTAGA |
| Cdk7-F | TGTGGAAAAGATCCGTGTCA |
| Cdk7-R | GTCACACGCTGGTATCGTTC |
| Med26-F | ATCGGGATCTTCGTGAACTG |
| Med26-R | GTGCATCTGCACAAAATGCT |
| Trl/GAGA-F | ATAGGACGCCGCTTGTATTG |
| Trl/GAGA-F | CAAGAGCGAAGGACAAAAGG |
| Nelf-E F | CGTTAATGGGCTCAATCTGG |
| Nelf-E R | TTGTTTCCTTCGCCAAACC |
| Primer- Neg Fwd (Pol II ChIP) | CCATTAATCGAGGGCTGAAA |

| | |
|---|---|
| Primer- Neg Rev (Pol II ChIP) | TTGGGGCATAAACAGAGGAC |
| Rpl19 H F | GAAATCGCCAATGCCAACT |
| Rpl19 H R | GGGAATGGACCGTCACAG |
| RnpS1 qPCR F | GAGAAGGTCGCCAAAGAGAA |
| RnpS1 qPCR R | CTGGAGTCGCTGCTGGAG |
| Box B Fwd KpnI | NNNGGTACCACTACCAAACTGGGGATTCCTG |
| Box B Rev KpnI | NNNGGTACCGACCTCGAGATAATATCCTCGATAG |
| Box B 5' F | gcccctcgactagtccaaat |
| Box B 5' R | ccgggcctttctttatgttt |
| Act 5' | acacaa agccgctcca tcag |
| Act5' F | cattgcggctgataaggttt |
| Act5' R | gctttgtgtcgggaggagta |
| Luc 5' F | taccaacatggccgaagac |
| Luc 5'R | ggaaccagggcgtatctctt |
| Luc 5' F 2 | ttgtttccaaaaaggggttg |
| Luc 5'R2 | acgtgtacatcgactgaaatcc |
| Luc GB F | tgggttacctaagggtgtgg |
| Luc GB R | aaaaccgtgatggaatggaa |
| Luc GB F 2 | tcgccaaaagcactctgatt |
| Luc GB R 2 | ctggaagatggaagcgtttt |
| Luc 3' F | gtaacaaccgcgaaaaagttg |
| Luc 3' R | gcccttcttggcctttatg |
| Luc 3' F2 | gggacgaagacgaacacttct |
| Luc 3' R2 | cgaagatgttggggtgttg |
| Ren 5' F | agtggtgggccagatgtaaa |
| Ren 5'R | aataagaagaggccgcgtta |
| Ren GB F | ttattgaatcggacccagga |
| Ren GB R | catcaggtgcatcttcttgc |
| Ren 3' F | ttattgaatcggacccagga |
| Ren 3' R | catcaggtgcatcttcttgc |
| Cdk9 qPCR Fwd | TCGTGTTCGCAGAAATCAAA |
| Cdk9 qPCR rev | TAAAGCACGAGAACGTGGTG |
| Mapk TSS-F | GGGGATGTTGCAGTCTTGTT |
| Mapk TSS-R | CCCCACGTAGTAACGCAGAT |
| Mapk-Exon2 F | TGCTGAAGTTATAAGGGGACAAA |
| Mapk-Exon2 R | CAACCATGCCATAAGCTCCT |
| Mapk-Exon3 F | CGGATGACACGCTAACAAAC |
| Mapk-Exon3 R | GACAATAAGTTTGGTGTTCAAAGG |
| Mapk-Exon4 F | TGGCCATTACTCCAAATACCA |
| Mapk-Exon4 R | CATAACGCGAATCACTTCCA |
| Mapk-Exon5 F | ACATTCATTCCGCAAACGTC |
| Mapk-Exon5 R | TTAAGTCGCACGTCTTGTTCA |
| Mapk-Exon6 F | CCCGATGGTATAGAGCACCT |
| Mapk-Exon6 R | AACATTTCAGCCAAAATGCAG |
| Mapk-Exon7 F | CTGGGCGAAACTATTTCCAA |
| Mapk-Exon7 R | CCTCGACAGGAATCCGTTTA |
| Mapk-Exon8 F | TTCTCGAGATGCCCTGAAGT |
| Mapk-Exon8 R | TTAAGGCGCATTGTCTGGTT |
| PolII Neg-Fwd | AGCAATGTCGCTTCACACAC |
| PolII Neg-Rev | CTTGCGCCTAAGCTATTTGG |
| Mapk Fwd SQ | TACGCCGTCGATTTTGATAAATC |
| Mapk Rev SQ | TTCTTTACTTTCTTTAATCGATCTTTAATATTC |
| BoxB 5' F Com | agggccctatcgaggatatt |
| Bbs8 5' Rev | GATGGACGGCCCAGAGTC |
| Bbs8 3' Fwd | GCCAAGTCCTACCTGAATGC |
| Crk 5' Rev | CCAGCTTTAAGAACACATTTTGA |
| Crk 3' Fwd | CAGATTTAAATGTAAGTGTTCCAAAAA |
| Piwi 5' Rev | TCGAAGACATTGTTTAATTTTGG |

| | |
|---|---|
| Piwi 3' Fwd | TGTTTGCGACATAACGGAAT |
| PolyA 3' Rev Com | ttatcatgtctggatcctcgat |

Primers used for the cloning experiments

| | |
|---|---|
| boxB-Fwd KpnI | NNNGGTACCACTACCAAACTGGGGATTCCTG |
| boxB-Rev StuI | NNNAGGCCTGCGGCCGCGACCTCGAGATAATATCCTCGATAG |
| piwi-Fwd NotI | NNNGCGGCCGCGTCACAACTTGAGTTGGCAC |
| piwi-Rev StuI | CCTGTGATGGGCCGGTATGTAAAC |
| Crk-Fwd Not I | NNNGCGGCCGCGACGTTTCTGATAGGAACAGGT |
| Crk-Rev StuI | CCTACTGTTTTACGCGGGCGTATG |
| BBS8-Fwd NotI | NNNGCGGCCGCGCAATTTGCAACGCATTGGC |
| BBS8-Rev StuI | CCTCTTATAGCTTGTAATGCACATCCATG |
| Mut Mago-Fwd | GCGTTGGGGCACGAATTCTTGGAGTT |
| Mut Mago-Rev | GGTGTCGTTCTTGTAGTTGGAGTTGTTGGC |
| Cdk9 Fwd-EcoRV | NNNNNGATATCATGGCGCACATGTCCCACATG |
| Cdk9 Rev-NotI | NNNNGCGGCCGCCTACCAAACCCGGTCAATCATACTG |
| Mago Fwd-EcoRV | GATATCATGTCCACGGAGGACTTTTACCTACG |
| Mago Rev-NotI | GCGGCCGCTTATATGGGCTTGATCTTGAAATGCAGG |
| elF4AIII Fwd EcoRV | ATCATGGCGCGCAAGAATGCCCAGGCGG |
| elFAIII Rev NotI | NNNNNGCGGCCGCTTAGATCAAGTCAGCCACGTTCATGGG |
| Y14 EcoRV Fwd | NNNNGATATCATGGCCGATGTGTTGGACATT |
| Y14 NotI Rev | NNNNGCGGCCGCTTATCTGCGACGCTTTTCGGA |
| RnpS1 EcoRV Fwd | NNNNGATATCATGGCGCGTGCCCAGAGTCT |
| RnpS1 NotI Rev | NNNNGCGGCCGCCTAACGAGAGCTGTCACTGCTATTG |
| eGFP EcoRV Fwd | NNNNGATATCATGGTGAGCAAGGGCGAGGA |
| eGFP NotI Rev | NNNNGCGGCCGCTTACTTGTACAGCTCGTCCATGCC |

Supplementary Table2: Number of genes used in the box plots

| Figure | Category | Number | |
|---|---|---|---|
| Fig 1h | Low | 919 | |
| | Mod Low | 919 | |
| | Mod High | 919 | |
| | High | 919 | |
| Fig 2f | Low | 88-EJC bound | 1863-Expressed |
| | Mod Low | 120-EJC bound | 1862-Expressed |
| | Mod High | 162-EJC bound | 1862-Expressed |
| | High | 247-EJC bound | 1863-Expressed |
| Fig 2g | Unbound | 4661 | |
| | pre-EJC bou | 816 | |
| Fig 3c | Unbound | 4661 | |
| | pre-EJC bou | 816 | |
| Fig 3e | Unbound | 4661 | |
| | pre-EJC bou | 816 | |
| Fig 4a, 4b | no-intron | 897 | |
| | 0-0.5kb | 3935 | |
| | 0.5kb-2kb | 1156 | |
| | 2-10kb | 910 | |
| | above 10kb | 372 | |
| Fig 4c | no-intron | 94 | |
| | 0-0.5kb | 1347 | |
| | 0.5kb-2kb | 1058 | |
| | 2-10kb | 1056 | |
| | above 10kb | 567 | |
| Fig 4d | no-intron | 200-EJC bound | 3742-Unbound |
| | 0-0.5kb | 129-EJC bound | 1200-Unbound |
| | 0.5kb-2kb | 151-EJC bound | 1056-Unbound |
| | 2-10kb | 108-EJC bound | 567-Unbound |
| | above 10kb | 29-EJC bound | 885-Unbound |
| Fig 5e | Unbound | 4661 | |
| | pre-EJC bou | 816 | |
| Fig 6f | Mago KD | 62 | |
| | Cdk9 KD | 47 | |
| | Mago, Cdk9 | 52 | |
| Fig S1f | Mago | | |
| | no-intron | 224 | |
| | 0-0.5kb | 553 | |
| | 0.5kb-2kb | 222 | |
| | 2-10kb | 358 | |
| | above 10kb | 279 | |
| | eIF4AIII | | |
| | no-intron | 249 | |
| | 0-0.5kb | 1308 | |
| | 0.5kb-2kb | 493 | |
| | 2-10kb | 534 | |

| | | | |
|---|---|---|---|
| | above 10kb | 325 | |
| | Y14 | | |
| | no-intron | 227 | |
| | 0-0.5kb | 1070 | |
| | 0.5kb-2kb | 323 | |
| | 2-10kb | 401 | |
| | above 10kb | 246 | |
| | Btz | | |
| | no-intron | 50 | |
| | 0-0.5kb | 143 | |
| | 0.5kb-2kb | 73 | |
| | 2-10kb | 132 | |
| | above 10kb | 90 | |
| | RnpS1 | | |
| | no-intron | 14 | |
| | 0-0.5kb | 44 | |
| | 0.5kb-2kb | 24 | |
| | 2-10kb | 33 | |
| | above 10kb | 13 | |
| Fig S3c | Ctrl KD | 65 | |
| | Mago KD | 65 | |
| Fig S8b | Quartile1 | 88-EJC bound | 1863-Expressed |
| | Quartile2 | 120-EJC bound | 1862-Expressed |
| | Quartile3 | 162-EJC bound | 1862-Expressed |
| | Quartile4 | 247-EJC bound | 1863-Expressed |
| Fig S8c | Quartile1 | 456-Nelf bound | 1407-Expressed |
| | Quartile2 | 780-Nelf bound | 1082-Expressed |
| | Quartile3 | 992-Nelf bound | 870-Expressed |
| | Quartile4 | 1229-Nelf bound | 634-Expressed |
| Fig S8d | Quartile1 | 268-EJC bound | 763-Unbound |
| | Quartile2 | 229-EJC bound | 801-Unbound |
| | Quartile3 | 149-EJC bound | 881-Unbound |
| | Quartile4 | 134-EJC bound | 897-Unbound |
| Fig S8d | Quartile1 | 715-Nelf bound | 204-Unbound |
| | Quartile2 | 640-Nelf bound | 279-Unbound |
| | Quartile3 | 544-Nelf bound | 375-Unbound |
| | Quartile4 | 515-Nelf bound | 404-Unbound |

## Supplementary References

1. Gilchrist, D.A. et al. Pausing of RNA polymerase II disrupts DNA-specified nucleosome organization to enable precise gene regulation. *Cell* **143**, 540-51 (2010).
