# Supplementary material for: Promoter-proximal pausing mediated by the exon junction complex regulates splicing
Source: Nat Commun. 2019 Jan 31;10:521. doi: 10.1038/s41467-019-08381-0 (PMC6355915; doi:10.1038/s41467-019-08381-0)
Supplement: Supplementary file 3 — Reporting Summary [file 41467_2019_8381_MOESM3_ESM.pdf]

nature research

Corresponding author(s): NCOMMS-18-14341B

Reporting Summary

Nature Research wishes to improve the reproducibility of the work that we publish. This form provides structure for consistency and transparency in reporting. For further information on Nature Research policies, see Authors & Referees and the Editorial Policy Checklist.

Statistical parameters

When statistical analyses are reported, confirm that the following items are present in the relevant location (e.g. figure legend, table legend, main text, or Methods section).

n/a	Confirmed	
☐	☒	The exact sample size (n) for each experimental group/condition, given as a discrete number and unit of measurement
☐	☒	An indication of whether measurements were taken from distinct samples or whether the same sample was measured repeatedly
☐	☒	The statistical test(s) used AND whether they are one- or two-sided<br>*Only common tests should be described solely by name; describe more complex techniques in the Methods section.*
☐	☒	A description of all covariates tested
☐	☒	A description of any assumptions or corrections, such as tests of normality and adjustment for multiple comparisons
☐	☒	A full description of the statistics including central tendency (e.g. means) or other basic estimates (e.g. regression coefficient) AND variation (e.g. standard deviation) or associated estimates of uncertainty (e.g. confidence intervals)
☐	☒	For null hypothesis testing, the test statistic (e.g. F, t, r) with confidence intervals, effect sizes, degrees of freedom and P value noted<br>*Give P values as exact values whenever suitable.*
☒	☐	For Bayesian analysis, information on the choice of priors and Markov chain Monte Carlo settings
☒	☐	For hierarchical and complex designs, identification of the appropriate level for tests and full reporting of outcomes
☐	☒	Estimates of effect sizes (e.g. Cohen's d, Pearson's r), indicating how they were calculated
☐	☒	Clearly defined error bars<br>*State explicitly what error bars represent (e.g. SD, SE, CI)*

Our web collection on statistics for biologists may be useful.

Software and code

Policy information about availability of computer code

Data collection	blc2fastq(v1.8.4/v2.19.1),bowtie2 (v2.2.8), MACS2 software (v2.1.1-20160309), STAR (v.2.5.1b)
Data analysis	R and Bioconductor package, ChIPseeker package on Bioconductor, htSeq count (v 0.6.1.p1), DEseq2 (v.1.10.1), DEXSeq (v.1.16.10), rMATS (v.3.2.1b), Deeptools (v.2.2.3), BEDOPS (v.2.4.30), damidseq_pipeline (GitHub), NGS.plot(v.2.61)

For manuscripts utilizing custom algorithms or software that are central to the research but not yet described in published literature, software must be made available to editors/reviewers upon request. We strongly encourage code deposition in a community repository (e.g. GitHub). See the Nature Research guidelines for submitting code & software for further information.

Data

Policy information about availability of data

All manuscripts must include a data availability statement. This statement should provide the following information, where applicable:

- Accession codes, unique identifiers, or web links for publicly available datasets
- A list of figures that have associated raw data
- A description of any restrictions on data availability

Datasets from RNA-Seq, ChIP-Seq, 4sU-Seq, MNase-Seq and DamID-Seq have been deposited in NCBI's Gene Expression Omnibus and are accessible through GEO

series accession number GSE92389. A Reporting Summary for this Article is available as a Supplementary Information file. All other data supporting the findings of this study are available from the corresponding author upon request.

Field-specific reporting

Please select the best fit for your research. If you are not sure, read the appropriate sections before making your selection.

☒ Life sciences ☐ Behavioural & social sciences ☐ Ecological, evolutionary & environmental sciences

For a reference copy of the document with all sections, see nature.com/authors/policies/ReportingSummary-flat.pdf

Life sciences study design

All studies must disclose on these points even when the disclosure is negative.

Sample size	All the experiments included in this study were performed in at least two independent biological replicates.
Data exclusions	No data was excluded from the study
Replication	All of the genomics studies were performed in set of two biological replicates. Other experiments were performed in triplicates with each replicates consisting of three technical replicates (qPCR etc). The precise number of replicates are also indicated in the respective figure legends.
Randomization	N/A.The randomization was not relevant for this study as we are looking into the effect of exon junction complex on transcription, we always performed the control and various knockdown conditions in one cohort to rule out the batch effect. The details about the experimental set up is provided in methods section.
Blinding	N/A. The blinding was also not relevant for this study as the biological groups/conditions were well defined.

Reporting for specific materials, systems and methods

Materials & experimental systems

n/a	Involved in the study	
☐	☒	Unique biological materials
☐	☒	Antibodies
☐	☒	Eukaryotic cell lines
☒	☐	Palaeontology
☐	☒	Animals and other organisms
☒	☐	Human research participants

Methods

n/a	Involved in the study	
☐	☒	ChIP-seq
☒	☐	Flow cytometry
☒	☐	MRI-based neuroimaging

Unique biological materials

Policy information about availability of materials

Obtaining unique materials	Mago antibody (described in methods section). The authors are willing to provide the antibody on request.

Antibodies

Antibodies used	Mago Antibody (in house generated), Pol II RBP1 (Diagenode-15200004), Ser2P (Abcam-ab5095), Ser5P (Chromotek-3E8), ARNA3 Pol II (Progen-65123), Ser2P(Chromotek-3E10), HA antibody (Santacruz-sc7392), anti Flag M2 (Sigma-F3165), hCdk9 (Santacruz-8338)
Validation	The dMago antibody was validated in our lab. The remaining antibodies are widely used and references describing their use are available on manufacturer's website.

Eukaryotic cell lines

Policy information about cell lines

Cell line source(s)	S2R+ cells from Drosophila Genomic Rescource Center, Hela Cells (a kind gift from Helle Ullrich lab)
Authentication	Tested for mycoplasma contamination
Mycoplasma contamination	Negative for mycoplasma contamination, test performed using luminence assay
Commonly misidentified lines (See ICLAC register)	*Name any commonly misidentified cell lines used in the study and provide a rationale for their use.*

Animals and other organisms

Policy information about studies involving animals; ARRIVE guidelines recommended for reporting animal research

Laboratory animals	Drosphila lines used in the study are described in the method section.
Wild animals	*Provide details on animals observed in or captured in the field; report species, sex and age where possible. Describe how animals were caught and transported and what happened to captive animals after the study (if killed, explain why and describe method; if released, say where and when) OR state that the study did not involve wild animals.*
Field-collected samples	*For laboratory work with field-collected samples, describe all relevant parameters such as housing, maintenance, temperature, photoperiod and end-of-experiment protocol OR state that the study did not involve samples collected from the field.*

ChIP-seq

Data deposition

☒ Confirm that both raw and final processed data have been deposited in a public database such as GEO.

☒ Confirm that you have deposited or provided access to graph files (e.g. BED files) for the called peaks.

Data access links
May remain private before publication.

https://www.ncbi.nlm.nih.gov/geo/query/acc.cgi?acc=GSE92389

Files in database submission

GEO Accession Number Name of the samples
GSM2428782 4SU-controlKD_NT_Rep1
GSM2428783 4SU-controlKD_t0_Rep1
GSM2428784 4SU-controlKD_t2_Rep1
GSM2428785 4SU-controlKD_t8_Rep1
GSM2428786 4SU-controlKD_t16_Rep1
GSM2428787 4SU-MagoKD_NT_Rep1
GSM2428788 4SU-MagoKD_t0_Rep1
GSM2428789 4SU-MagoKD_t2_Rep1
GSM2428790 4SU-MagoKD_t8_Rep1
GSM2428791 4SU-MagoKD_t16_Rep1
GSM2428792 4SU-controlKD_NT_Rep2
GSM2428793 4SU-controlKD_t0_Rep2
GSM2428794 4SU-controlKD_t2_Rep2
GSM2428795 4SU-controlKD_t8_Rep2
GSM2428796 4SU-controlKD_t16_Rep2
GSM2428797 4SU-MagoKD_NT_Rep2
GSM2428798 4SU-MagoKD_t0_Rep2
GSM2428799 4SU-MagoKD_t2_Rep2
GSM2428800 4SU-MagoKD_t8_Rep2
GSM2428801 4SU-MagoKD_t16_Rep2
GSM2428833 Input_HA_Ctrl
GSM2428834 Input_HA_Mago
GSM2428835 Input_HAMago_MagoKD
GSM2428836 HA_Ctrl_Rep1
GSM2428837 HA_Ctrl_Rep2
GSM2428838 HA_Ctrl_RNAseT1_Rep1
GSM2428839 HA_Ctrl_RNAseT1_Rep2
GSM2428840 HA_Mago_Rep1
GSM2428841 HA_Mago_Rep2
GSM2428842 HA_Mago_RNAseT1_Rep1
GSM2428843 HA_Mago_RNAseT1_Rep2
GSM2428844 HAMago_MagoKD_Rep1
GSM2428845 HAMago_MagoKD_Rep2
GSM2428857 Input_HA_RnpS1

GSM2428858 HA_RnpS1_Rep1
GSM2428859 HA_RnpS1_Rep2
GSM2428860 HA_RnpS1_RNAseT1_Rep1
GSM2428861 HA_RnpS1_RNAseT1_Rep2
GSM2428886 polyA-mRNA_Cdk9KD_Rep1
GSM2428887 polyA-mRNA_Cdk9KD_Rep2
GSM2428888 polyA-mRNA_eIF4AIII_Rep1
GSM2428889 polyA-mRNA_eIF4AIII_Rep2
GSM2428890 polyA-mRNA_MagoKD_Rep1
GSM2428891 polyA-mRNA_MagoKD_Rep2
GSM2428892 polyA-mRNA_Cdk9-MagoDKD_Rep1
GSM2428893 polyA-mRNA_Cdk9-MagoDKD_Rep2
GSM2428894 polyA-mRNA_RnpS1KD_Rep1
GSM2428895 polyA-mRNA_RnpS1KD_Rep2
GSM2428896 polyA-mRNA_controlKD_Rep1
GSM2428897 polyA-mRNA_controlKD_Rep2
GSM2428898 polyA-mRNA_Y14KD_Rep1
GSM2428899 polyA-mRNA_Y14KD_Rep2
GSM2430688 HelacontrolKD_Input
GSM2430689 Hela-tot-PolII_controlKD_Rep1
GSM2430690 Hela-tot-PolII_controlKD_Rep2
GSM2430693 HelaMagohKD_Input
GSM2430694 Hela-tot-PolII_MagohKD_Rep1
GSM2430695 Hela-tot-PolII_MagohKD_Rep2
GSM2430951 MagoKD_Ser2P_Rep1
GSM2430952 MagoKD_Ser2P_Rep2
GSM2430959 Control KD_Ser2P_Rep1
GSM2430960 Control KD_Ser2P_Rep2
GSM2430963 Input_MagoKD_Ser2P
GSM2430964 MNAse_MagoKD_Rep1
GSM2430965 MNAse_MagoKD_Rep2
GSM2430966 Input_controlKD_Ser2P
GSM2430967 MNAse_controlKD_Rep1
GSM2430968 MNAse_controlKD_Rep2
GSM2857406 Control KD_K4Me3_Rep1
GSM2857407 Control KD_K4Me3_Rep2
GSM2857408 Mago KD_K4Me3_Rep1
GSM2857409 Mago KD_K4Me3_Rep2
GSM2857410 HA_eIF4AIII_Rep1
GSM2857411 HA_control_Rep1
GSM2857412 HA_control_Rep2
GSM2857413 HA_eIF4AIII_Rep2
GSM2857414 Input_HA-control
GSM2857415 Input_HA-eIF4AIII
GSM2857416 Input_HA-Y14
GSM2857417 HA_Y14_Rep1
GSM2857418 HA_Y14_Rep2
GSM2857419 4SU-frag_controlKD_Rep2
GSM2857420 4SU-frag_controlKD_Rep1
GSM2857421 4SU-frag_MagoKD_Rep1
GSM2857422 4SU-frag_MagoKD_Rep2
GSM2857423 tot-PolII_BtzKD_Rep1
GSM2857424 tot-PolII_BtzKD_Rep2
GSM2857425 tot-PolII_controlKD_Rep1
GSM2857426 tot-PolII_controlKD_Rep2
GSM2857429 tot-PolII_eIF4AIIIKD_Rep1
GSM2857430 tot-PolII_eIF4AIIIKD_Rep2
GSM2857431 Input_BtzKD
GSM2857432 Input_controlKD
GSM2857433 Input_eIF4AIIIKD
GSM2857434 Input_MagoKD
GSM2857436 Input_Y14KD
GSM2857437 tot-PolII_MagoKD_Rep1
GSM2857438 tot-PolII_MagoKD_Rep2
GSM2857443 tot-PolII_Y14KD_Rep1
GSM2857444 tot-PolII_Y14KD_Rep2
GSM2857445 B_tot-PolII_controlKD_Rep1
GSM2857446 B_tot-PolII_controlKD_Rep2
GSM2857447 B_tot-PolII_MagoKD_Rep1
GSM2857448 B_tot-PolII_MagoKD_Rep2
GSM2857449 B_tot-PolII_RnpS1KD_Rep1
GSM2857450 B_tot-PolII_RnpS1KD_Rep2
GSM2857451 polyA-mRNA_BtzKD_Rep1
GSM2857452 polyA-mRNA_BtzKD_Rep2
GSM2857455 B_polyA-mRNA_controlKD_Rep1

GSM2857456 B_polyA-mRNA_controlKD_Rep2
GSM3005249 Ctrl_Cdk9_Dam_1
GSM3005250 Ctrl_Cdk9_Dam_2
GSM3005251 Ctrl_Dam_1
GSM3005252 Ctrl_Dam_2
GSM3005253 Mago_Cdk9_Dam_1
GSM3005254 Mago_Cdk9_Dam_2
GSM3005255 Mago_Dam_1
GSM3005256 Mago_Dam_2
GSM3102403 B_Input_controlKD
GSM3102404 B_Input_MagoKD
GSM3102405 B_Input_RnpS1KD
GSM3103313 Input_ControlKD_K4Me3
GSM3103314 Input_MagoKD_K4Me3
GSM3417729 Input_HA_Ctrl [earlier passage]
GSM3417730 Input_HA_Mago [earlier passage]
GSM3417731 Input_HAMago_MagoKD [earlier passage]
GSM3417732 HA_Ctrl_Rep1 [earlier passage]
GSM3417733 HA_Ctrl_Rep2 [earlier passage]
GSM3417734 HA_Ctrl_RNAseT1_Rep1 [earlier passage]
GSM3417735 HA_Ctrl_RNAseT1_Rep2 [earlier passage]
GSM3417736 HA_Mago_Rep1 [earlier passage]
GSM3417737 HA_Mago_Rep2 [earlier passage]
GSM3417738 HA_Mago_RNAseT1_Rep1 [earlier passage]
GSM3417739 HA_Mago_RNAseT1_Rep2 [earlier passage]
GSM3417740 HAMago_MagoKD_Rep1 [earlier passage]
GSM3417741 HAMago_MagoKD_Rep2 [earlier passage]
GSM3417742 Input_Mago KD
GSM3417743 Mago Pol II_1
GSM3417744 Mago Pol II_2
GSM3417745 Input_ Ctrl KD
GSM3417746 Ctrl Pol II_1
GSM3417747 Ctrl Pol II_2

Genome browser session
(e.g. UCSC)

No longer applicable

Methodology

Replicates

All of the genomics studies were performed in set of two biological replicates. The precise number of replicates are indicated in the figure legends.

Sequencing depth

ChIP samples were amplified by PCR through 13 cycles. Samples were then processed for 45bp paired-end sequencing. ChIP-seq with 18-25 million reads for each sample.

Antibodies

HA tagged ChIP- anti-HA (Santacruz, sc7392)
Pol II ChIP- RBP1 (Diagenode, 15200004)
Ser2P ChIP- anti Ser2CTD (Abcam, ab5095)

Peak calling parameters

macs2 callpeak -m 10 50 -g dm -f BAMPE -q 0.05 --keep-dup auto -bw <mean insert size> [Pol II ChIP]
macs2 callpeak -m 10 50 -g dm -f BAMPE -q 0.05 --fe-cutoff 2.0 --keep-dup auto -bw <mean insert size> [HA-ChIP]

Data quality

Peaks were controlled with 5% FDR and also with replicate reproducibility, and peak were also checked for normalized strand coefficient (NSC). The details about the methods are provided in the method section.

Software

de-multiplexing was performed using blc2fastq(v1.8.4/v2.19.1). Reads were aligned using bowtie2 (v2.2.8) using dm6 (BDGP6, ensemble release 84) and hg38 (ensemble release 84). Peaks were called using MACS2 software (v2.1.1-20160309).
